# Lp-PLA_2_, scavenger receptor class B type I gene (*SCARB1*) rs10846744 variant, and cardiovascular disease

**DOI:** 10.1371/journal.pone.0204352

**Published:** 2018-10-05

**Authors:** Ani Manichaikul, Xin-Qun Wang, Li Li, Jeanette Erdmann, Guillaume Lettre, Joshua C. Bis, Dawn Waterworth, Mary Cushman, Nancy S. Jenny, Wendy S. Post, Walter Palmas, Michael Y. Tsai, Lars Wallentin, Harvey White, Heribert Schunkert, Christopher J. O’Donnell, David M. Herrington, Stephen S. Rich, Michelle L. O’Donoghue, Annabelle Rodriguez

**Affiliations:** 1 Center for Public Health Genomics, University of Virginia, Charlottesville, VA, United States of America; 2 Department of Public Health Sciences, Biostatistics Section, University of Virginia, Charlottesville, VA, United States of America; 3 Genomic Medicine, PAREXEL International, Durham, NC, United States of America; 4 Institut für Integrative und Experimentelle Genomik, University of Lübeck, Lübeck, Germany; 5 DZHK (German Research Centre for Cardiovascular Research), partner site Hamburg, Kiel, Lübeck, Germany; 6 Montreal Heart Institute, Montreal, Quebec, Canada; 7 Université de Montréal, Montreal, Quebec, Canada; 8 Cardiovascular Health Research Unit, University of Washington, Seattle, WA, United States of America; 9 Department of Medicine, University of Washington, Seattle, WA, United States of America; 10 Genetics, GlaxoSmithKline, King of Prussia, PA, United States of America; 11 Department of Medicine, University of Vermont, Burlington, VT, United States of America; 12 Department of Pathology and Laboratory Medicine, University of Vermont, Burlington, VT, United States of America; 13 Division of Cardiology, Department of Medicine, Johns Hopkins University School of Medicine and Department of Epidemiology, Johns Hopkins Bloomberg School of Public Health, Baltimore, MD, United States of America; 14 Division of General Medicine, Department of Medicine, Columbia University College of Physicians & Surgeons, New York, NY, United States of America; 15 Department of Laboratory Medicine and Pathology, University of Minnesota, Minneapolis, MN, United States of America; 16 Uppsala Clinical Research Center and Department of Medical Sciences, Uppsala University, Uppsala, Sweden; 17 Auckland City Hospital Green Lane Cardiovascular Sciences, Auckland, New Zealand; 18 DZHK (German Research Centre for Cardiovascular Research), partner site Munich Heart Alliance, Munich, Germany; 19 Deutsches Herzzentrum München, Technische Universität München, Munich, Germany; 20 Cardiology Section, Boston Veteran’s Administration Healthcare, Boston, MA, United States of America; 21 NHLBI and Boston University Framingham Heart Study, Framingham, MA, United States of America; 22 Department of Internal Medicine, Wake Forest University School of Medicine, Winston-Salem, NC, United States of America; 23 TIMI Study Group, Cardiovascular Division, Brigham and Women's Hospital, Boston MA, United States of America; 24 Department of Cell Biology, Center for Vascular Biology, University of Connecticut Health, Farmington, CT, United States of America; Universitatsklinikum Hamburg-Eppendorf, GERMANY

## Abstract

**Background:**

We previously reported association of *SCARB1* SNP rs10846744 with common carotid IMT (cIMT) and cardiovascular disease (CVD) events. Since rs10846744 has been reported in association with Lp-PLA_2_ mass and activity, we hypothesized that inflammatory pathways might mediate the association of rs10846744 with atherosclerosis.

**Methods:**

We first examined association of rs10846744 in CVD in multiple large-scale consortium-based genome-wide association studies. We further examined 27 parameters of interest, including Lp-PLA_2_ mass and activity, inflammatory markers, and plasma phospholipid fatty acids, and fatty acid ratios in participants from the Multi-Ethnic Study of Atherosclerosis (MESA), as potential mediators in the pathway linking rs10846744 with cIMT and incident CVD. Finally, we examined the association of rs10846744 with Lp-PLA_2_ activity, cardiovascular outcomes, and interaction with the Lp-PLA_2_ inhibitor, darapladib, in the Stabilization of Atherosclerotic Plaque by Initiation of Darapladib Therapy (STABILITY) and Stabilization of Plaque using Darapladib-Thrombolysis in Myocardial Infarction 52 (SOLID-TIMI 52) studies.

**Results:**

*SCARB1* rs10846744 was associated with coronary artery disease events in CARDIoGRAMplusC4D (odds ratio 1.05; 95% CI [1.02, 1.07]; *P* = 1.4x10^-4^). In combined analysis across race/ethnic groups in MESA, rs10846744 was associated with Lp-PLA_2_ mass (*P* = 0.04) and activity (*P* = 0.001), homocysteine (*P* = 0.03), LDL particle number (*P* = 0.01), docosahexaenoic acid [DHA] (*P* = 0.01), docosapentaenoic acid [DPA] (*P* = 0.04), DPA/ eicosapentaenoic acid [EPA] ratio (*P* = 0.002), and DHA/EPA ratio (*P* = 0.008). Lp-PLA_2_ activity was identified as a mediator of rs10846744 with cIMT in a basic model (*P* = 8x10^-5^), but not after adjustment for CVD risk factors. There was no interaction or modifier effect of the Lp-PLA_2_ inhibitor darapladib assignment on the relationship between rs10846744 and major CVD events in either STABILITY or SOLID-TIMI 52.

**Summary:**

*SCARB1* rs10846744 is significantly associated with Lp-PLA_2_ activity, atherosclerosis, and CVD events, but Lp-PLA_2_ activity is not a mediator in the association of rs10846744 with cIMT in MESA.

## Introduction

In this era of genome wide association studies (GWAS), there is a need to identify the causal pathways of significant single nucleotide polymorphisms (SNP) on disease phenotypes. We showed that the scavenger receptor class B type I gene (*SCARB1)* intronic rs10846744 SNP was significantly associated with subclinical atherosclerosis (SCA) as measured by common carotid intima-media thickness (cIMT) in participants from the Multi-Ethnic Study of Atherosclerosis (MESA) [1]. In the full MESA cohort and in replication studies, we showed that rs10846744 was significantly associated with SCA and cardiovascular disease (CVD) events [2]. The association of rs10846744 with SCA and CVD events remained significant after multivariable regression analysis that included total, LDL and HDL cholesterol and particle sizes, age, sex, race, body mass index (BMI), hypertension, smoking, diabetes mellitus, renal disease, and lipid lowering medications. Thus, we hypothesized that factors other than lipids and other traditional cardiovascular risk factors might mediate the relationship between *SCARB1* SNP rs10846744 and risk of atherosclerotic disease.

Suchindran *et al*. [3] had performed a GWAS of lipoprotein-associated phospholipase A_2_ (Lp-PLA_2_) mass and activity in participants from the Framingham Heart Study, and identified rs10846744 as being positively associated with Lp-PLA_2_ mass and activity. Grallert *et al*. [4] reported that rs10846744 was positively associated with Lp-PLA_2_ activity but not Lp-PLA_2_ mass in an expanded GWAS from the CHARGE consortium. In agreement to what we observed, Kleber *et al*. [5] showed that Lp-PLA_2_ mass predicted total and cardiovascular mortality independently of known CV risk factors. These results led us to the refined hypothesis that an inflammatory and/or fatty-acid related pathway might be causal in the association of rs10846744 with atherosclerotic disease.

In this study we sought to (i) validate the association of rs10846744 with SCA and CV events from consortium-based GWAS, (ii) examine associations of rs10846744 with Lp-PLA_2_ mass and activity, inflammatory markers and fatty acids in MESA, and (iii) perform formal mediation analyses to quantify the role of selected covariates as mediators of the association of rs10846744 with cIMT in MESA. We expanded our analyses to examine the association between rs10846744 and Lp-PLA_2_ activity and CV events from the STABILITY and SOLID-TIMI 52 trials, in addition to the interaction with the Lp-PLA_2_ inhibitor darapladib [6,7].

## Methods

**Fig 1:**
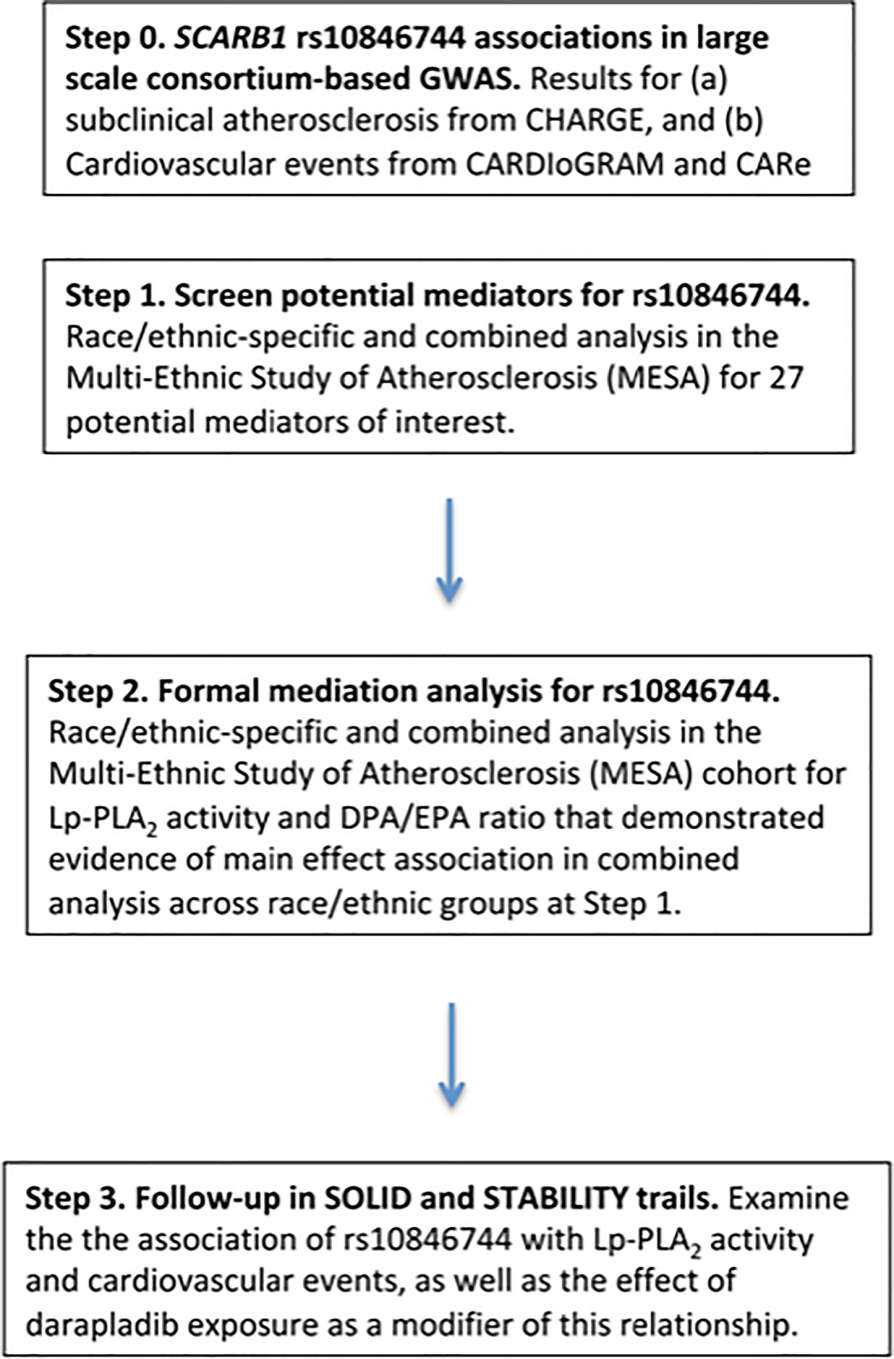
Overview of approach.

We present an overview of our approach in **Fig 1**. Detailed Methods are provided below.

### SCA and CAD from GWAS cohorts

We examined the association of rs10846744 with SCA in a GWAS of cIMT, internal carotid IMT [iIMT] and plaque in cohorts of European ancestry from the CHARGE consortium [8]. We examined the association of rs10846744 with coronary heart disease (CHD) in results from the GWAS of African-American cohorts in CARe [9] and with coronary artery disease (CAD) in cohorts of various ancestries from the CARDIoGRAMplusC4D consortium [10]. *CHARGE*: GWAS analyses on IMT were performed by meta-analysis of ~31,000 individuals from nine participating studies within Cohorts for Heart and Aging Research in Genomic Epidemiology (CHARGE) [11]. *CARe*: African-American participants for the GWAS were drawn from five population-based studies: Atherosclerosis Risk in Communities, Coronary Artery Risk Development in young Adults, Cleveland Family Study, Jackson Heart Study, and MESA [9]. Genetic analysis of CHD in CARe included 881 cases and 6682 controls *CARDIoGRAMplusC4D*: The Coronary ARtery DIsease Genome-wide Replication And Meta-Analysis (CARDIoGRAM) plus The Coronary Artery Disease (C4D) Genetics) consortium combined data from GWAS on >60,000 CAD cases and >123,000 controls representing primarily European ancestry (77% of participants), with other race/ethnic groups represented [10].

### MESA study design

MESA is a longitudinal study of SCA and risk factors that predict progression to clinically overt CVD or progression of the subclinical disease [12]. The first clinic visits occurred in 2000–2002 in 6,814 participants recruited from six field centers across the United States, and all participants were free of CVD at the baseline exam. Approximately 38% of the recruited participants were Caucasian, 28% African-American, 22% Hispanic, and 12% Asian, predominantly of Chinese descent, with race/ethnicity classified based on participant self-report (**Table 1**). One ancillary study (MESA Family Study, MESAFS) recruited family members of African-American and Hispanic participants, specifically for genetic studies. Another ancillary study (MESA Air) evaluated the effects of air pollution on atherosclerosis risk [13].

**Table 1:** Characteristics of MESA, MESA family and MESA Air participants across four ethnic groups.

Participant characteristics*	Caucasian	African- American	Hispanic	Chinese-American
No. subjects	n = 2470	n = 2507	n = 2071	n = 758
Women	1189 (48.1)	1101 (43.9)	954 (46.1)	372 (49.1)
Age, years	63.0 [54.0, 71.0]	60.0 [53.0, 68.0]	60.0 [52.0, 68.0]	62.0 [53.0, 71.0]
BMI, kg/m^2^	27.1 [24.2, 30.4]	29.4 [26.1, 33.7]	28.6 [25.9, 32.0]	23.7 [21.7, 26.0]
Education: completed high school	2342 (95.1) (n = 2462)	2239 (89.8) (n = 2492)	1135 (54.8) (n = 2071)	580 (75.1) (n = 757)
Education: completed technical degree, associate degree, bachelor's degree or higher	1492 (60.6)	1229 (49.3)	438 (21.1)	390 (50.5)
Diabetes (yes/no)	145 (5.9) (n = 2464)	431 (17.3) (n = 2493)	381 (18.4) (n = 2068)	103 (13.6) (n = 756)
Serum creatinine, mg/dl	0.9 [0.8, 1.1] (n = 2464)	1.0 [0.8, 1.1] (n = 2492)	0.9 [0.8, 1.0] (n = 2068)	0.9 [0.7, 1.0] (n = 756)
LDL-C, mg/dl	115.0 [96.0, 136.0] (n = 2430)	116.0 [95.0, 137.0] (n = 2481)	118.0 [96.0, 139.0] (n = 2024)	114.0 [96.0, 131.2] (n = 744)
HDL-C, mg/dl	50.0 [41.0, 61.0] (n = 2462)	50.0 [42.0, 61.0] (n = 2495)	46.0 [39.0, 55.0] (n = 2070)	48.0 [40.0, 56.0] (n = 757)
Hypertension (yes/no)	954 (38.6) (n = 2470)	1503 (60.0) (n = 2507)	862 (41.6) (n = 2071)	287 (37.9) (n = 758)
Ever smoke (yes/no)	1375 (55.8) (n = 2463)	1327 (53.2) (n = 2494)	939 (45.3) (n = 2070)	188 (24.8) (n = 757)
Current smoke (yes/no)	285 (11.5) (n = 2470)	489 (19.5) (n = 2507)	283 (13.7) (n = 2070)	42 (5.5) (n = 758)
Lipid medication (yes/no)	445 (18.0) (n = 2469)	464 (18.5) (n = 2504)	345 (16.7) (n = 2070)	108 (14.2) (n = 758)
Clinical events*	n = 2467	n = 1602	n = 1426	n = 758
CV-All (yes/no)	313 (12.7)	177 (11.0)	173 (12.1)	64 (8.4)
CV-Hard (yes/no)	210 (8.5)	131 (8.2)	136 (9.5)	39 (5.1)
Death (all-cause) (yes/no)	369 (15.0)	274 (17.1)	186 (13.0)	76 (10.0)
Common carotid IMT, mm	0.84 [0.73, 0.97] (n = 2445)	0.86 [0.75, 0.99] (n = 2465)	0.81 [0.71, 0.93] (n = 2056)	0.81 [0.71, 0.92] (n = 755)
Internal carotid IMT, mm	0.89 [0.71, 1.38] (n = 2421)	0.91 [0.70, 1.30] (n = 2436)	0.84 [0.68, 1.20] (n = 2019)	0.73 [0.60, 0.94] (n = 751)
**Biomarkers:**				
Lp-PLA_2_ mass, ng/ml	188.1 [164.8, 213.5] (n = 1968)	162.2 [136.5, 188.1] (n = 1166)	175.8 [153.9, 198.5] (n = 1138)	163.8 [136.8, 187.4] (n = 643)
Lp-PLA_2_ activity, nmol/min/ml	153.0 [129.1, 179.1] (n = 1981)	134.4 [112.8, 158.3] (n = 1208)	151.2 [127.2, 172.8] (n = 1159)	154.7 [127.2, 177.6] (n = 645)
hsCRP, mg/L	1.8 [0.8, 4.1] (n = 2459)	2.4 [1.1, 5.4] (n = 1913)	2.4 [1.1, 4.8] (n = 1687)	0.9 [0.5, 1.8] (n = 756)
IL-6, pg/ml	1.1 [0.7, 1.7] (n = 2423)	1.4 [0.9, 2.1] (n = 1533)	1.4 [0.9, 2.1] (n = 1387)	0.9 [0.6, 1.3] (n = 748)
Homocysteine, μmol/L	8.7 [7.4, 10.4] (n = 2466)	8.9 [7.5, 10.9] (n = 1598)	8.5 [7.2, 10.3] (n = 1426)	8.4 [7.1, 10.3] (n = 757)
PAI-1, ng/ml	19.0 [9.0, 34.0] (n = 423)	16.0 [8.0, 33.0] (n = 181)	20.5 [11.0, 39.2] (n = 216)	24.0 [12.0, 39.0] (n = 96)
E-selectin, ng/ml	47.7 [34.0, 59.7] (n = 435)	52.9 [41.6, 71.2] (n = 188)	58.4 [43.7, 77.3] (n = 221)	49.1 [31.0, 65.0] (n = 98)
sICAM-1, ng/ml	274.9 [241.2, 313.0] (n = 1176)	247.7 [174.0, 306.6] (n = 421)	285.8 [244.9, 328.4] (n = 554)	226.7 [203.3, 256.1] (n = 290)
LDL particle number, nmol/L	1223.0 [1015.8, 1445.0] (n = 2464)	1181.0 [972.5, 1439.0] (n = 1595)	2.7 [1.1, 4.0] (n = 1422)	1178.5 [968.8, 1396.8] (n = 758)
**Fatty acids:**	n = 2394	n = 1533	n = 1378	n = 751
ALA, %	0.2 [0.1, 0.2]	0.1 [0.1, 0.2]	0.2 [0.1, 0.2]	0.2 [0.1, 0.2]
EPA, %	0.7 [0.5, 1.0]	0.7 [0.5, 1.0]	0.5 [0.4, 0.7]	0.9 [0.6, 1.5]
DHA, %	3.2 [2.5, 4.2]	4.1 [3.3, 5.0]	2.9 [2.3, 3.8]	5.0 [4.1, 5.9]
DPA, %	0.9 [0.8, 1.1]	0.9 [0.8, 1.1]	0.9 [0.7, 1.0]	0.9 [0.8, 1.1]
LA, %	19.8 [17.9, 21.7]	18.9 [17.1, 20.9]	20.9 [18.9, 23.1]	23.1 [20.8, 25.3]
GLA, %	0.1 [0.1, 0.1]	0.1 [0.1, 0.1]	0.1 [0.1, 0.1]	0.1 [0.1, 0.1]
DGLA, %	3.1 [2.6, 3.7]	2.9 [2.5, 3.3]	3.6 [3.0, 4.1]	2.7 [2.2, 3.3]
AA, %	11.3 [9.7, 12.8]	13.2 [11.6, 14.8]	10.9 [9.3, 12.8]	10.3 [8.9, 11.8]
*SCARB1* rs10846744 frequency of effect allele C (vs. reference allele G)	0.18	0.62	0.35	0.57

Data are presented as n (%) for binary measures or median [IQR] for continuous measure.

*Clinical events are reported for MESA Classic participants only and reflect a smaller sample size compared to other measures. CV-All includes suspected and adjudicated cases; CV-Hard includes only adjudicated cases.

### Phenotyping of MESA participants

#### SCA and CV

The measures of SCA included ultrasound measurements of cIMT and iIMT [14]. Cardiovascular events were adjudicated by a MESA committee [15]. CV events included incident myocardial infarction (MI), definite angina, probable angina (if followed by coronary artery bypass grafting and percutaneous coronary intervention), resuscitated cardiac arrest, stroke, stroke death, coronary heart disease death, or other CV death. We examined probable or confirmed CV events described as CV-All, confirmed CV events described as CV-Hard, incident MI, and all-cause mortality.

#### Lp-PLA_2_ mass and activity

Lp-PLA_2_ mass and activity were measured by diaDexus Inc. (South San Francisco, CA, USA) [16].

#### Inflammatory markers

Six inflammatory markers were selected for the association studies: interleukin-6 (IL-6), high-sensitivity C-reactive protein (hsCRP), plasminogen activator inhibitor-1 (PAI-1), soluble intercellular adhesion molecule-1 (sICAM-1), E-selectin, and homocysteine. Blood samples were collected at baseline and stored at –80°C until analysis. Interleukin-6, hsCRP, and PAI-1 levels were measured at the Laboratory for Clinical Biochemistry Research (University of Vermont, Burlington, VT) while homocysteine was measured at the University of Minnesota. The samples were processed using a standardized protocol from the Cardiovascular Health Study (CHS) [17]. Plasma IL-6, sICAM-1, and E-selectin were measured using quantitative enzyme-linked immunosorbent assays (Quantikine HS Human IL-6 Immunoassay, Parameter Human sICAM-1 Immunoassay, Parameter Human sE-Selectin Immunoassay, respectively; R&D Systems, Minneapolis, MN). PAI-1 levels were measured by ELISA (Diagnostica Stago, Inc., Parsippany, NJ). Plasma homocysteine levels were measured using a fluorescence polarization immunoassay (IMx homocysteine assay, Axis Biochemicals ASA, Oslo, Norway) with the IMx analyzer (Abbott Diagnostics, Abbott Park, IL). *Fatty acids*: Phospholipid fatty acids were extracted from EDTA plasma [18,19]. Lipids were extracted from the plasma using a chloroform/methanol extraction method, and the cholesterol esters, triglyceride, phospholipids and free fatty acid fractions were separated by thin layer chromatography. Fatty acids from the phospholipid fractions were derivatized to methyl esters and then injected on a gas chromatograph equipped with a 100m capillary column, and detected by flame ionization. The fatty acids detected were expressed as a percent of total fatty acids.

#### Genotyping

All MESA participants were genotyped on the Affymetrix 6.0 array which included the rs10846744 SNP. Details are provided in **S1 File**.

### Statistical analysis in MESA

We examined the association of rs10846744 with Lp-PLA_2_ mass and activity, hsCRP, homocysteine, IL-6, E-selectin, PAI-1, sICAM-1, LDL particle number, n-3 fatty acids (α-linolenic acid [ALA], eicosapentaenoic acid [EPA], docosahexaenoic acid [DHA], docosapentaenoic acid [DPA]) and n-6 fatty acids (linoleic acid [LA], gamma-linoleic acid [GLA], dihomo-gamma-linoleic acid [DGLA], arachidonic acid [AA]), in addition to 10 different fatty acid ratios (listed in **Table 2**). We performed linear regression of quantitative phenotypes or logistic regression of dichotomous phenotypes in R [20]. Fixed effect meta-analysis was performed to combine estimated effects and standard errors from stratified analyses, as implemented in METAL [21]. We further implemented trans-ethnic meta-analysis using MANTRA [22]. We also report Heterogeneity I-squared and Heterogeneity P-values from Cochran’s Q test as implemented in METAL [21].

**Table 2:** Results of associations between rs10846744 and the mediation factors within MESA race/ethnic groups.

Mediator	Group	N	Beta	SE	*P*-value	log10 Bayes Factor	Heterogeneity I-squared / Heterogeneity *P*-value
Lp-PLA_2_ mass, ng/ml	Caucasian	1842	2.934	1.665	0.08		
	African-American	1155	-1.252	1.728	0.47		
	Hispanic	1130	3.224	1.561	0.04		
	Chinese-American	602	1.781	2.358	0.45		
	Meta-analysis		1.775	0.882	0.04	0.30	32.0 / 0.22
Lp-PLA_2_ activity, nmol/min/ml	Caucasian	1861	1.541	1.356	0.26		
	African-American	1202	1.469	1.366	0.28		
	Hispanic	1155	3.573	1.384	0.01		
	Chinese-American	603	4.328	2.206	0.05		
	Meta-analysis		2.424	0.744	**0.001**	**1.69**	0.0 / 0.50
hsCRP, mg/L	Caucasian	2452	0.009	0.042	0.84		
	African-American	1949	0.001	0.039	0.99		
	Hispanic	1739	0.055	0.039	0.17		
	Chinese-American	701	0.022	0.055	0.69		
	Meta-analysis		0.022	0.021	0.31	-0.25	0.0 / 0.78
Homocysteine, μmol/L	Caucasian	2305	-0.007	0.010	0.46		
	African-American	1585	-0.011	0.010	0.26		
	Hispanic	1418	-0.014	0.010	0.19		
	Chinese-American	703	-0.019	0.013	0.16		
	Meta-analysis		-0.012	0.005	0.03	0.69	0.0 / 0.90
IL-6, pg/ml	Caucasian	2277	-0.010	0.025	0.68		
	African-American	1530	-0.005	0.025	0.85		
	Hispanic	1387	0.007	0.025	0.77		
	Chinese-American	695	0.013	0.036	0.73		
	Meta-analysis		-0.001	0.013	0.97	-0.80	0.0 / 0.94
E-selectin, ng/ml	Caucasian	551	-0.482	1.598	0.76		
	African-American	223	-0.679	2.406	0.78		
	Hispanic	270	-1.478	2.418	0.54		
	Chinese-American	93	-0.763	3.843	0.84		
	Meta-analysis		-0.760	1.116	0.50	-0.40	0.0 / 0.99
PAI-1, ng/ml	Caucasian	398	0.033	0.094	0.73		
	African-American	180	0.159	0.095	0.10		
	Hispanic	216	-0.046	0.085	0.59		
	Chinese-American	90	-0.092	0.116	0.43		
	Meta-analysis		0.019	0.048	0.70	-0.40	18.8 / 0.30
sICAM-1, ng/ml	Caucasian	1249	5.421	3.252	0.10		
	African-American	452	-6.193	6.483	0.34		
	Hispanic	600	-3.829	4.686	0.41		
	Chinese-American	260	-3.125	5.187	0.55		
	Meta-analysis		-0.371	2.230	0.87	-0.35	36.1 / 0.20
LDL particle number, nmol/L	Caucasian	2311	8.388	12.148	0.49		
	African-American	1585	9.345	12.942	0.47		
	Hispanic	1418	41.361	13.901	0.003		
	Chinese-American	705	12.628	17.364	0.47		
	Meta-analysis		17.354	6.862	0.01	1.16	24.7 / 0.26
ALA, %	Caucasian	2247	0.034	0.013	0.01		
	African-American	1525	0.008	0.013	0.56		
	Hispanic	1373	-0.008	0.015	0.58		
	Chinese-American	698	0.014	0.021	0.49		
	Meta-analysis		0.013	0.007	0.08	0.23	36.4 / 0.19
EPA, %	Caucasian	1600	0.041	0.025	0.09		
	African-American	891	0.009	0.027	0.74		
	Hispanic	692	0.002	0.029	0.94		
	Chinese-American	51	-0.097	0.147	0.51		
	Meta-analysis		0.019	0.015	0.23	-0.14	0.0 / 0.60
DHA, %	Caucasian	2247	0.088	0.045	0.05		
	African-American	1522	0.039	0.049	0.42		
	Hispanic	1369	0.051	0.042	0.23		
	Chinese-American	696	0.091	0.078	0.24		
	Meta-analysis		0.063	0.025	0.01	0.86	0.0 / 0.86
DPA. %	Caucasian	2244	0.000	0.008	0.97		
	African-American	1521	0.018	0.008	0.03		
	Hispanic	1370	0.009	0.008	0.29		
	Chinese-American	694	0.011	0.013	0.43		
	Meta-analysis		0.009	0.004	0.04	0.53	0.0 / 0.47
LA, %	Caucasian	2254	-0.049	0.113	0.67		
	African-American	1530	-0.084	0.105	0.42		
	Hispanic	1376	-0.129	0.121	0.29		
	Chinese-American	697	-0.290	0.191	0.13		
	Meta-analysis		-0.107	0.062	0.08	0.24	0.0 / 0.74
GLA, %	Caucasian	2238	0.000	0.002	0.98		
	African-American	1525	0.003	0.002	0.10		
	Hispanic	1368	0.000	0.002	0.82		
	Chinese-American	691	0.003	0.003	0.35		
	Meta-analysis		0.001	0.001	0.24	-0.35	0.0 / 0.60
DGLA, %	Caucasian	2252	-0.010	0.031	0.75		
	African-American	1526	0.004	0.025	0.88		
	Hispanic	1377	0.031	0.035	0.38		
	Chinese-American	699	0.051	0.044	0.24		
	Meta-analysis		0.012	0.016	0.45	-0.46	0.0 / 0.64
AA, %	Caucasian	2255	-0.041	0.089	0.64		
	African-American	1527	-0.071	0.089	0.43		
	Hispanic	1375	0.105	0.096	0.28		
	Chinese-American	698	-0.007	0.118	0.95		
	Meta-analysis		-0.008	0.048	0.88	-0.50	0.0 / 0.57
AA/LA	Caucasian	2246	0.000	0.006	0.10		
	African-American	1524	0.000	0.007	0.97		
	Hispanic	1369	0.012	0.007	0.06		
	Chinese-American	696	0.005	0.008	0.48		
	Meta-analysis		0.004	0.003	0.20	-0.19	0.0 / 0.55
GLA/LA	Caucasian	2244	0.000	0.001	0.81		
	African-American	1524	0.001	0.001	0.08		
	Hispanic	1372	0.000	0.001	0.64		
	Chinese-American	696	0.000	0.001	0.65		
	Meta-analysis		0.001	0.000	0.12	-0.54	0.0 / 0.86
DGLA/GLA	Caucasian	2250	-0.011	0.016	0.51		
	African-American	1525	-0.025	0.015	0.09		
	Hispanic	1374	0.008	0.017	0.63		
	Chinese-American	698	0.009	0.025	0.73		
	Meta-analysis		-0.009	0.009	0.33	-0.28	0.0 / 0.45
AA/DGLA	Caucasian	2251	-0.002	0.012	0.86		
	African-American	1527	-0.008	0.012	0.51		
	Hispanic	1373	0.005	0.012	0.70		
	Chinese-American	697	-0.020	0.019	0.29		
	Meta-analysis		-0.004	0.007	0.54	-1.09	0.0 / 0.70
DGLA/LA	Caucasian	2248	0.000	0.002	0.94		
	African-American	1525	0.001	0.002	0.46		
	Hispanic	1371	0.002	0.002	0.35		
	Chinese-American	697	0.004	0.002	0.10		
	Meta-analysis		0.002	0.001	0.11	0.22	0.0 / 0.53
DHA/ALA	Caucasian	2250	-0.010	0.018	0.58		
	African-American	1529	0.001	0.018	0.95		
	Hispanic	1375	0.025	0.021	0.23		
	Chinese-American	698	-0.011	0.027	0.69		
	Meta-analysis		-0.002	0.010	0.87	-0.40	0.0 / 0.60
EPA/ALA	Caucasian	2248	-0.002	0.022	0.94		
	African-American	1525	0.025	0.023	0.27		
	Hispanic	1370	0.032	0.023	0.16		
	Chinese-American	697	0.044	0.042	0.30		
	Meta-analysis		0.020	0.012	0.10	-0.54	0.0 / 0.65
DPA/EPA	Caucasian	2248	-0.020	0.009	0.03		
	African-American	1527	-0.012	0.010	0.20		
	Hispanic	1376	-0.010	0.010	0.33		
	Chinese-American	697	-0.025	0.015	0.11		
	Meta-analysis		-0.016	0.005	0.002	**1.52**	0.0 / 0.78
DHA/EPA	Caucasian	2248	-0.024	0.017	0.17		
	African-American	1526	-0.030	0.019	0.12		
	Hispanic	1374	-0.022	0.022	0.31		
	Chinese-American	696	-0.051	0.034	0.148		
	Meta-analysis		-0.028	0.011	0.008	1.09	0.0 / 0.90
DHA/DPA	Caucasian	2253	0.023	0.012	0.06		
	African-American	1526	-0.009	0.012	0.43		
	Hispanic	1376	0.004	0.013	0.77		
	Chinese-American	697	-0.001	0.015	0.92		
	Meta-analysis		0.004	0.006	0.52	-0.50	20.6 / 0.29

Regression models were adjusted for age, sex, study site, and PCs of ancestry. Genetic association shown for rs10846744 effect allele C (vs. reference allele G).

Meta-analysis results were obtained as follows: (a) *P*-values from fixed effects meta-analysis implemented in METAL [21], (b) log10 Bayes factors in favor of association from trans-ethnic meta-analysis implemented in MANTRA [22], (c) Heterogeneity I-squared and Heterogeneity P-values from Cochran’s Q test as implemented in METAL [21]. We have highlighted in **bold** fixed effects meta-analysis *P*-values reaching the Bonferroni threshold of α*≤0.05/27 traits≤0.0019, and trans-ethnic meta-analysis log10 Bayes factors>1.5

For potential mediators demonstrating a statistically significant main association with rs10846744 in meta-analysis across race/ethnic groups (based on fixed effects meta-analysis p-value reaching the Bonferroni threshold of α*≤0.05/27 traits≤0.0019, or a trans-ethnic meta-analysis log10 Bayes factor of association > 1.5), we proceeded to perform mediation analysis by performing formal comparisons of regression models with and without the mediators of interest. Details are provided in **S1 File**.

### STABILITY and SOLID-TIMI 52 studies

We examined the association of rs10846744 with Lp-PLA_2_ activity, cardiovascular outcomes, and interaction with the Lp-PLA_2_ inhibitor, darapladib, in the Stabilization of Atherosclerotic Plaque by Initiation of Darapladib Therapy (STABILITY) and Stabilization of Plaque using Darapladib-Thrombolysis in Myocardial Infarction 52 (SOLID-TIMI 52) studies.

STABILITY was a multinational double-blind trial that randomly assigned 15,828 subjects with stable CHD to either once-daily darapladib or placebo therapy for a median follow-up period of 3.7 years [6]. The primary endpoint for STABILITY was time to CV death, MI or stroke. In STABILITY, multivariable regression models included adjustment for age, sex, region, BMI, hyperlipidemia, statin use, baseline LDL, baseline HDL, eGFR, smoking, diabetes, prior MI, principal components (PCs) of ancestry and randomized treatment arm.

The SOLID-TIMI 52 was a multinational, double-blind trial that enrolled 13,026 participants who had been hospitalized with an acute coronary syndrome in the past 30 days and randomized them to once daily darapladib or placebo for a median follow-up of 2.5 years [7]. The primary endpoint was CHD death, MI or urgent coronary revascularization. In SOLID-TIMI 52, multivariable regression models included adjustment for age, sex, region, BMI, hyperlipidemia, statin use, baseline LDL, baseline HDL, eGFR, smoking, diabetes, prior MI, index diagnosis (STEMI vs not), days from qualifying event, PCs of ancestry and randomized treatment arm.

Both STABILITY and SOLID-TIMI 52 included participants of Caucasian, African-American, Asian and other race/ethnicities, as determined by participant self-report. In STABILITY and SOLID-TIMI 52, meta-analysis used random effects models across all race/ethnic groups. Genotype data were generated on the HumanOmniExpressExome-8 v1 array for STABILITY and the Axiom Biobank Plus Genotyping Array with custom content array for SOLID. Genotype imputation was performed using the 1000 Genomes Project phase I reference panel for both studies using MACH/minimac. *SCARB1* rs10846744 was directly genotyped in SOLID-TIMI 52 and well imputed in STABILITY (imputation R^2^ = 0.95).

### Human subjects approval

All of the studies for which we conducted data analyses in this manuscript, including MESA, STABILITY, SOLID-TIMI 52, as well as those included in the consortium-based GWAS studies from CHARGE, CARe and CARDIoGRAMplusC4D were approved by their respective institutional review boards and with written informed consent from the study participants. In particular, our work on analysis of primary data from MESA was approved by the University of Virginia Institutional Review Board for Health Sciences Research.

## Results

### Association of rs10846744 with SCA and CHD in GWAS

In CARDIoGRAMplusC4D [10], rs10846744 effect allele C (vs. reference allele G). was significantly association with CAD (n cases = 60,801, n controls = 123,504; odds ratio 1.05; 95% CI [1.02, 1.07]; *P* = 1.4x10^-4^). In the CHARGE GWAS of SCA in Caucasians [8], there was no association between rs10846744 and cIMT (n = 23,442, *P* = 0.90), iIMT (n = 6,046, *P* = 0.28) or carotid plaque (n = 17,222, *P* = 0.99). In the CARe GWAS of CHD in African-Americans [9], there was no significant association of rs10846744 with CHD (n cases = 881, n controls = 6682, *P* = 0.53). We did not observe a significant association of rs10846744 with cIMT in CHARGE nor with CHD events in CARe.

### MESA demographics

The MESA and MESA Family participants included 2,470 Caucasian, 2,507 African-American, and 2,071 Hispanic and 758 Chinese-American individuals, roughly evenly distributed between males and females (**Table 1**). Clinical events were assessed after a median 12.1 years of follow-up. The prevalence of probable or confirmed CV events (CVD-All) was 12.7%, 11.0%, 12.1%, and 8.4% in Caucasians, African-Americans, Hispanics and Chinese-Americans, respectively, while the prevalence of confirmed CVD events was 8.5%, 8.2%, 9.5% and 5.1%, respectively (**Table 1**). Consistent with our previous report [23], we observed consistent directions of effect across race/ethnic groups for the association of cIMT with rs10846744, with little evidence of heterogeneity across groups (**S1 Table,** Heterogeneity I-squared = 0.0, Heterogeneity *P*-value = 0.68). In contrast, the association of rs10846744 with cardiovascular events showed considerable heterogeneity across groups (**S1 Table**, CVD-confirmed Heterogeneity I-squared = 81.6, Heterogeneity *P*-value = 0.001).

### Association of rs10846744 with Lp-PLA_2_ mass and activity

**Fig 2:**
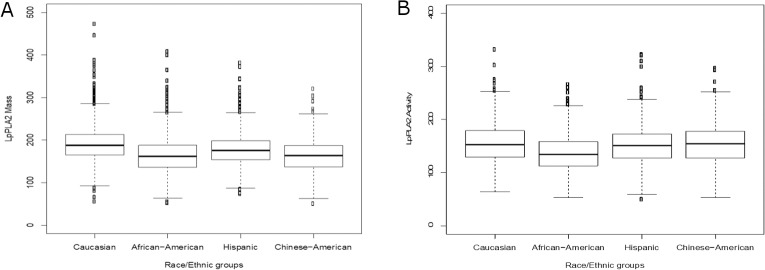
Lp-PLA_2_ mass and activity levels stratified by race in MESA. (A). Lp-PLA_2_ mass was measured using the PLAC^TM^ test (17). In Caucasians, Lp-PLA_2_ mass was 188.1 [164.8–213.5] ng/ml (median [IQR], N = 1968); African-Americans, 162.2 [136.5–188.1], N = 1166; Hispanic, 175.8 [153.9–198.5], N = 1138; and Chinese-Americans, 163.8 [136.8–187.4], N = 643. (B). Lp-PLA_2_ activity was measured by a radiometric assay using tritium-labeled platelet activating factor [^3^H]PAF as the substrate (17). In Caucasians, median Lp-PLA_2_ activity was 153.0 [129.1–179.1] nmol/min/ml (median [IQR], N = 1981); African-Americans, 134.4 [112.8–158.3], N = 1208; Hispanic, 151.2 [127.2–172.8], N = 1159; and Chinese-Americans, 154.7 [127.2–177.6], N = 645. Kruskal-Wallis rank sum test indicated that there were significant overall differences in either Lp-PLA_2_ mass or activity across race/ethnic groups (*P* = 1.2×10^−88^ and *P* = 3.1×10^−49^ respectively). Wilcoxon rank sum test indicated that in Caucasians and Hispanics Lp-PLA_2_ mass was significantly higher than in African-Americans and Chinese-Americans (Bonferroni corrected *P* = 4.9×10^−67^ and *P* = 1.4×10^−46^, respectively), while for Lp-PLA_2_ activity, Caucasians, Hispanics, and Chinese-Americans had significantly higher levels than African-Americans (Bonferroni corrected *P* = 1.2×10^−47^,1.3×10^−19^, 4.7×10^−48^, respectively).

Lp-PLA_2_ mass and activity were different between the race/ethnic groups, with the largest difference observed between Caucasians (median [interquartile range] for Lp-PLA_2_ mass, 188.1 [164.8, 213.5]; Lp-PLA_2_ activity, 153.0 [129.1, 179.1] nmol/min/ml) and African-Americans (Lp-PLA_2_ mass 162.2 [136.5, 188.1] ng/ml; Lp-PLA_2_ activity, 134.4 [112.8, 158.3] nmol/min/ml, Bonferroni corrected for all pairwise race/ethnic comparisons *P* = 4.9×10^−67^ and *P =* 1.2×10^−47^) (**Fig 2A and 2B**). Lp-PLA_2_ mass and activity were positively correlated in Caucasians (r = 0.92), Hispanics (r = 0.98) and Chinese-Americans (r = 0.44), but inversely correlated in African-Americans (r = -0.40).

We examined the association of rs10846744 with Lp-PLA_2_ mass and activity in MESA participants. Meta-analysis across race/ethnic groups revealed a significant association of rs10846744 with Lp-PLA_2_ activity (*P* = 0.001), Lp-PLA_2_ mass (*P* = 0.04), (**Table 2**).

### Association of rs10846744 with inflammatory biomarkers in MESA

We selected the inflammatory biomarkers and fatty acids based on their availability in the MESA database as well as their well-characterized roles in CVD. Meta-analysis across race/ethnic groups revealed association between rs10846744 and DPA/EPA ratio in trans-ethnic meta-analysis with a log10 Bayes factor = 1.52. No additional parameters under investigation demonstrated statistically significant association in fixed effects meta-analysis based on our Bonferroni threshold of α*≤0.05/27 traits≤0.0019, nor in trans-ethnic meta-analysis based on our threshold of log10 Bayes factor < 1.5. We observed nominal associations between rs10846744 and homocysteine (*P* = 0.03), LDL particle number (*P* = 0.01), DHA (*P* = 0.01), DPA (*P* = 0.04), and DHA/EPA (*P* = 0.008) (**Table 2**).

### Mediation analysis of inflammatory and/or fatty acids in the association of rs10846744 with cIMT and incident CVD in MESA

Based on the results of main effect association (Table 2), Lp-PLA_2_ activity and DHA/EPA ratio were carried forward for mediation analysis. Using the bias-corrected bootstrap method, a meta-analysis resulted in Lp-PLA_2_ activity, but not DHA/EPA, as a mediator in the association of rs10846744 with cIMT (*P* = 0.00008) in a model adjusted for age, sex, study site, and PCs of ancestry (**Table 3**). In a fully adjusted model, Lp-PLA_2_ activity was no longer a significant mediator (**Table 3**).

**Table 3:** Mediation analysis of inflammatory biomarkers and fatty acids in the association of rs10846744 with cIMT in MESA: Basic and fully adjusted models.

**BASIC MODEL**							
**Mediation variable**	**Coefficient of rs10846744**	**Coefficient of rs10846744**	**Difference in coefficients**	**Bias-Corrected Bootstrap**			
	**Model 1**	**Model 2**	**Model 2 –Model 1**	**95% CI**		***P*-value**	
**Caucasian:**							
Lp-PLA_2_ activity (n = 1838)	0.01104	0.01066	-0.00038	(-0.00183, 0.00011)		0.12	
DPA/EPA (n = 2217)	0.01055	0.01037	-0.00018	(-0.00122, 0.00045)		0.49	
**African-American:**							
Lp-PLA_2_ activity (n = 1154)	0.01400	0.01363	-0.00037	(-0.00338, 0.00247)		0.81	
DPA/EPA (n = 1464)	0.01557	0.01555	-0.00002	(-0.00082, 0.00052)		0.81	
**Hispanic:**							
Lp-PLA_2_ activity (n = 1049)	0.01922	0.01715	-0.00207	(-0.00482, -0.00050)		0.01	
DPA/EPA (n = 1237)	0.02202	0.02171	-0.00031	(-0.00155, 0.00022)		0.24	
**Chinese-American:**							
Lp-PLA_2_ activity (n = 599)	0.01574	0.01476	-0.00099	(-0.00421, 0.00038)		0.15	
DPA/EPA (n = 693)	0.01516	0.01489	-0.00027	(-0.00242, 0.00080)		0.46	
**Meta-analysis:**			**Effect**	**StdErr**		**P-value**	**Heterogeneity** **I-squared /** **Heterogeneity** ***P*-value**
Lp-PLA_2_ activity			-0.00120	0.0003		**7.5e-05**	3.7 / 0.37
DPA/EPA			-0.00010	0.0002		0.65	0.0 / 0.90
**FULLY ADJUSTED MODEL**							
**Variable**	**Coefficient of rs10846744**	**Coefficient of rs10846744**	**Difference in coefficients**	**Bias-Corrected Bootstrap**			
	**Model 1**	**Model 2**	**Model 2 –Model 1**	**95% CI**	***P*-value**		
**Caucasian:**							
Lp-PLA_2_ activity (n = 1805)	0.00949	0.00968	0.00019	(-0.00030, 0.00143)	0.32		
DPA/EPA (n = 2179)	0.01088	0.00995	-0.00024	(-0.00132, 0.00039)	0.35		
**African-American:**							
Lp-PLA_2_ activity (n = 1135)	0.0112	0.01111	-0.00009	(-0.00131, 0.00039)	0.50		
DPA/EPA (n = 1439)	0.01278	0.01289	0.00011	(-0.00045, 0.00109)	0.50		
**Hispanic:**							
Lp-PLA_2_ activity (n = 1027)	0.01612	0.01545	-0.00067	(-0.00251, 0.00011)	0.11		
DPA/EPA (n = 1029)	0.01836	0.01811	-0.00025	(-0.00156, 0.00020)	0.24		
**Chinese-American:**							
Lp-PLA_2_ activity (n = 590)	0.01466	0.01486	0.00021	(-0.00122, 0.00212)	0.63		
DPA/EPA (n = 679)	0.01666	0.01675	0.00009	(-0.00117, 0.00184)	0.71		
**Meta-analysis:**			**Effect**	**StdErr**	***P*-value**		**Heterogeneity** **I-squared /** **Heterogeneity** ***P*-value**
Lp-PLA_2_ activity			-0.00001	0.0003	0.85		27.6 / 0.25
DPA/EPA			-0.00010	0.0002	0.71		0.0 / 0.48

Basic model: Regression models were adjusted for age, sex, study site, and PCs of ancestry; Unlike model 2, model 1 did not include the mediation variable. DPA/EPA was square root transformed. Genetic association shown for rs10846744 effect allele C (vs. reference allele G). For meta-analysis results, Heterogeneity I-squared and Heterogeneity *P*-values from Cochran’s Q test are reported as implemented in METAL [21].

Fully adjusted model: Regression models were fully adjusted for age, sex, BMI, diabetes, creatinine, LDL, HDL, Lipid medication, hypertension, ever smoke (yes/no), current smoke (yes/no), education, study site, and PCs of ancestry; Unlike model 2, model 1 did not include the mediation variable. DPA/EPA was square root transformed. Genetic association shown for rs10846744 effect allele C (vs. reference allele G). For meta-analysis results, Heterogeneity I-squared and Heterogeneity P-values from Cochran’s Q test are reported as implemented in METAL [21].

### Association of rs10846744 with CV events in STABILITY and SOLID-TIMI 52

We examined whether the Lp-PLA_2_ inhibitor darapladib would modify the association of rs10846744 with CVD events in the STABILITY and SOLID-TIMI 52 studies (**Table 4**). In STABILITY, meta-analysis showed an association of rs10846744 with baseline Lp-PLA_2_ activity (*P* = 7.2x10^-11^). When all subjects were pooled (n = 13,522), we observed an association of the rs10846744 SNP with major cardiovascular events (MCE: composite of coronary heart disease death, MI, or urgent coronary revascularization for myocardial ischemia) (*P* = 0.04). We did not observe a significant association of rs10846744 with major adverse cardiovascular events (MACE) that was defined as CV death, MI, or stroke. We did not observe an interaction effect between Lp-PLA_2_ activity or darapladib assignment and rs10846744 on CVD outcomes. In SOLID-TIMI 52, we did observe significant associations of rs10846744 with baseline Lp-PLA_2_ activity (all subjects pooled *P* = 1.18x10^-4^, meta-analysis *P* = 5.68x10^-2^). We did not observe significant associations between rs10846744 and CV outcomes (MCE or MACE), and neither were there any interactions between rs10846744 and Lp-PLA_2_ activity or darapladib assignment.

**Table 4:** Association of rs10846744 with CV events in STABILITY and SOLID-TIMI 52 studies. In STABILITY, multivariable regression models included adjustment for age, sex, region, BMI, hyperlipidemia, statin use, baseline LDL, baseline HDL, eGFR, smoking, diabetes, prior MI, principal components (PCs) of ancestry and randomized treatment arm. In SOLID-TIMI 52, multivariable regression models included adjustment for age, sex, region, BMI, hyperlipidemia, statin use, baseline LDL, baseline HDL, eGFR, smoking, diabetes, prior MI, index diagnosis (STEMI vs not), days from qualifying event, PCs of ancestry and randomized treatment arm. Genetic associations are shown for rs10846744 effect allele C (vs. reference allele G).

**STABILITY**						**SOLID-TIMI 52**					
***SCARB1 rs10846744 effects on LpPLA***_***2***_ ***activity at baseline***						***SCARB1 rs10846744 effects on LpPLA***_***2***_ ***activity at baseline***					
**Group**	**MAF**	**N**	**Beta**	**SE**	**P-value**	**Group**	**MAF**	**N**	**Beta**	**SE**	**P-value**
Caucasian	0.17	9448	4.15	0.67	5.52E-10	Caucasian	0.16	7503	3.305	0.76	1.51E-05
African-American	0.53	254	4.78	4.12	2.47E-01	African-American	0.59	185	5.823	4.44	1.91E-01
Asian	0.64	1257	3.18	2.06	1.22E-01	Asian	0.62	408	-2.661	3.51	4.49E-01
Other	0.25	1637	2.1	1.63	1.96E-01	Other	0.27	1303	0.977	1.63	5.50E-01
All subjects pooled	0.24	12596	3.57	0.59	1.49E-09	All subjects pooled	0.21	9399	2.581	0.67	1.18E-04
Meta-analysis		12596	3.82	0.59	7.22E-11	Meta-analysis		9399	2.209	1.16	5.68E-02
***SCARB1 rs10846744 survival analysis for MCE***						***SCARB1 rs10846744 survival analysis for MCE***					
**Group**	**MAF**	**N (events)**	**Beta**	**SE**	**P-value**	**Group**	**MAF**	**N (events)**	**Beta**	**SE**	**P-value**
Caucasian	0.17	9889(945)	0.0926	0.06	1.30E-02	Caucasian	0.16	8136(1137)	-0.017	0.06	7.70E-01
African-American	0.53	265(38)	-0.051	0.27	8.50E-01	African-American	0.59	208(52)	0.1072	0.24	6.60E-01
Asian	0.64	1641(101)	0.0894	0.15	5.60E-01	Asian	0.62	424(35)	0.1801	0.29	5.30E-01
Other	0.25	1727(180)	0.1906	0.13	1.30E-01	Other	0.27	1421(227)	-0.087	0.011	4.20E-01
All subjects pooled	0.24	13522(1264)	0.1053	0.05	4.00E-02	All subjects pooled	0.21	10189(1451)	-0.017	0.05	7.30E-01
***Darapladib treatment as a modifier of rs10846744 effects in survival analysis of MCE***						***Darapladib treatment as a modifier of rs10846744 effects in survival analysis of MCE***					
**Group**	**Regression Term**	**N (events)**	**Beta**	**SE**	**P-value**	**Group**	**Regression Term**	**N (events)**	**Beta**	**SE**	**P-value**
All subjects pooled	SNP main effect	13522(1264)	0.0108	0.14	0.94	All subjects pooled	SNP main effect	10189(1451)	-0.112	0.14	0.43
(for each trial)	Darapladib treatment	13522(1264)	0.0451	0.15	0.76	(for each trial)	Darapladib treatment	10189(1451)	-0.057	0.15	0.71
	Treatment x SNP	13522(1264)	0.0643	0.09	0.47		Treatment x SNP	10189(1451)	0.0639	0.09	0.47

In both STABILITY and SOLID-TIMI 52, meta-analysis used random effects models across all race/ethnic groups.

## Discussion

Our previous findings of a significant association of rs10846744 with CVD events in MESA were consistent with findings in CARDIoGRAMplusC4D and STABILITY. Nonetheless, we did not observe a significant association of rs10846744 with cIMT in CHARGE or CARe. These discrepancies do not argue against the influence of this enhancer region on CV phenotypes but for the urgent need to thoroughly evaluate the effect of linkage disequilibrium structure and gene-environment influences on CVD outcomes across different race/ethnic groups.

The rationale for examining the association of Lp-PLA_2_ mass and activity in the association of rs10846744 with SCA and incident CVD was based on the fact that in MESA traditional risk factors, including cholesterol levels, did not attenuate this significant association. In MESA participants, meta-analysis revealed a large positive effect size and significant association of Lp-PLA_2_ mass and activity with rs10846744. Based on race/ethnic stratification, we found that the large effect size and significance between rs10846744 and Lp-PLA_2_ mass and activity was observed in Hispanics. In Chinese-Americans we observed significance in the association of rs10846744 with Lp-PLA_2_ activity, while in Caucasians we observed borderline significance with Lp-PLA_2_ mass. Katan *et al*. [24] also examined race/ethnic differences in the association of Lp-PLA_2_ mass and activity with stroke in participants of the Northern Manhattan Stroke Study (NOMAS), with the majority of participants classified as Hispanics and female. They observed significant association of Lp-PLA_2_ mass with large artery atherosclerotic stroke in non-Hispanic Whites but not in African-Americans or Hispanics; these investigators did not stratify results based on the rs10846744 SNP, which might offer an explanation for the differences in our study results.

PhospholipaseA_2_ is a phospholipase that can be secreted into the circulation (secretory Lp-PLA_2_) or becomes lipoprotein associated (Lp-PLA_2_), with the latter mostly bound to LDL [25]. It was not too surprising that we identified a strong main effect of LDL particle numbers in the association with rs10846744, and this significant association was only observed in Hispanics. We did not observe significant main effects of HDL or its subfractions. Interestingly, of the other parameters we examined, fatty acids were found to have significant main effects in association with rs10846744, although beta effects were small and at times in opposing directions.

Mediation analysis was performed to determine if Lp-PLA_2_ activity was a causal factor in the association of rs10846744 with SCA. With the use of the bias-corrected bootstrap statistical analysis, and a meta-analysis using a model adjusting for age, sex, study site, and PCs of ancestry, we found that Lp-PLA_2_ activity was a significant mediator; however, in a fully adjusted model, Lp-PLA_2_ activity was not a significant mediator. This result suggests that one or a combination of the covariates in the fully adjusted model influences the mediation effect of Lp-PLA_2_ activity in the association of rs10846744 with SCA. Interestingly, Holmes et al. has addressed a research hypothesis similar to ours using Mendelian randomization studies to examine the role of secretory PLA2 (sPLA2) isoenzymes in CHD [26,27] and has not identified evidence for a causal role of these sPLA2 isoenzymes in CHD.

There has been a longstanding interest in the role of Lp-PLA_2_ mass and/or activity as a causal factor in atherosclerosis based on its biological role in lipid oxidation and epidemiological studies showing significant associations of Lp-PLA_2_ mass and/or activity with CAD [28–30]. However, recent clinical trials with darapladib failed to show benefit on secondary prevention of major coronary events [6,7]. In both STABILITY and SOLID, when stratified by the rs10846744 genotype, we observed significant association of this SNP with Lp-PLA_2_ activity (Lp-PLA_2_ mass was not ascertained in STABILTY or SOLID-TIMI 52). In STABILITY, we observed a significant association of rs10846744 in survival analyses for MCE, but this association was not modified by darapladib. That we observed an association of rs10846744 with major cardiovascular events in STABILITY but not in SOLID-TIMI 52 might be due to the higher prevalence of MI at baseline in STABILITY subjects as compared with SOLID-TIMI 52 participants. In addition, SOLID-TIMI 52 was a study of subjects with acute MI and the samples used for Lp-PLA_2_ activity were obtained, on average, at 14 days of hospitalization for the MI event. These factors might also explain the differences in the association of rs10846744 with major coronary events between STABILITY and SOLID-TIMI 52.

In summary, we provide strong evidence for the association of rs10846744, which resides within an enhancer region, with Lp-PLA_2_ activity and evidence from a number of large genetic studies for association with CVD events. It has been shown that traditional CV risk factors do not explain the association of rs10846744 and Lp-PLA_2_ with CV events. Mediation analysis revealed that Lp-PLA_2_ activity was a significant mediator in the association of rs10846744 with cIMT but only in a basic adjusted model. The fully adjusted model suggests that other factor(s) remain to be identified in this inflammatory pathway. In agreement with Polfus *et al*. [31], it appears that Lp-PLA_2_ activity is a biomarker for inflammation but it is not a primary mediator for CVD events.

## Supporting information

S1 File Supplementary methods. (DOCX)

S1 Table Results of associations between *SCARB1* rs10846744 and the primary outcomes within MESA race/ethnic groups. (DOCX)
