# Supplementary material for: Lp-PLA2, scavenger receptor class B type I gene (SCARB1) rs10846744 variant, and cardiovascular disease
Source: PLoS One. 2018 Oct 5;13(10):e0204352. doi: 10.1371/journal.pone.0204352 (PMC6173398; doi:10.1371/journal.pone.0204352)
Supplement: S1 File — (DOCX) [file pone.0204352.s001.docx]

**S1 File. Supplementary Methods for “Lp-PLA_2_, Scavenger Receptor Class B Type I Gene (*SCARB1*) rs10846744 variant, and Cardiovascular Disease” by Manichaikul *et al.***

**Genotyping in MESA.**

Participants recruited for the original MESA cohort (n=6,814) were genotyped in 2009 using the Affymetrix Human SNP array 6.0 through the NHLBI CARe (Candidate gene Association Resource, dbGaP Study Accession: phs000283.v5.p3) and SHARe (SNP Health Association Resource, dbGaP Study Accession: phs000209.v2.p1) projects [1]. We filtered individuals on SNP level call rate < 95%, and filtered SNPs call rate < 95%, heterozygosity > 53%, and removed all monomorphic SNPs. The genotypic data was deposited with MESA phenotypic data into dbGaP as the MESA SHARe project (study accession phs000209) for consenting individuals with 897,981 SNPs passing study specific quality control.

**Genetic association analysis in MESA.**

We examined the association of rs10846744 with Lp-PLA_2_ mass and activity, hsCRP, homocysteine, IL-6, sICAM-1, E-selectin, PAI-1, n-3 fatty acids (α-linolenic acid [ALA], eicosapentaenoic acid [EPA], docosahexaenoic acid [DHA], docosapentaenoic acid [DPA]) and n-6 fatty acids (linoleic acid [LA], gamma-linoleic acid [GLA], dihomo-gamma-linoleic acid [DGLA], arachidonic acid [AA]). The following continuous phenotypes were log-transformed prior to genetic analyses: IL-6, hsCRP, homocysteine, PAI-1, and ALA. Principal components of ancestry (PCs) were computed within race/ethnic groups [1]. To select individuals to be included in analysis, we stratified the full MESA cohort by ethnic group, and eliminated those individuals with top PCs > 3.5 SD from the mean within any race/ethnic group. For each of the phenotypic analyses, we then restricted the data set to individuals with data available for the particular trait of interest. For quantitative traits, outliers were defined as individuals with trait values more than 3.5 SD from the mean, with the mean and SD calculated separately for each of the stratified analyses performed.

For stratified analysis of Caucasian and Chinese-American cohorts, and for phenotypes in which there were insufficient families with phenotypes among African-American and Hispanics, an unrelated subset of individuals was constructed by selecting (at most) one individual from each pedigree based on inferred relationships in KING [2], and performed linear regression of quantitative phenotypes or logistic regression of dichotomous phenotypes in R [3]. For analysis of phenotypes with a substantial familial component among African-American and Hispanic cohorts, we performed analysis using an additive model with a linear mixed-effects model for quantitative traits, or generalized estimating equations for dichotomous traits, as implemented in the package R/GWAF [4]. In all analyses, a basic model including age, sex, study site, and PCs was used. Based on examination of PCs within each ethnic group, 3 PCs were used for analysis of Caucasians, 1 PC for African-Americans, 3 PCs for Hispanics, and 1 PC for Chinese-Americans.

**Statistical mediation analysis in MESA.**

For potential mediators demonstrating a significant main association with rs10846744 (under α*≤0.05/16≤0.003 after Bonferroni correction), we proceeded to perform formal mediation analysis. Examining cIMT as an outcome, the criteria for performing mediation analysis within MESA race/ethnic groups was based on (1) defining the set of participants for whom the phenotypic outcome, mediator of interest, genotypes, and additional covariates for adjustment had no missing data; (2) performing linear (or logistic) regression for the phenotypic outcome for the participants selected in (1) to estimate the effect of rs10846744 with adjustment for age, sex, study site, PCs of ancestry, BMI, diabetes status (yes/no, defined as combining treated diabetes and the 2003 ADA fasting criterion [5]), serum creatinine, LDL-C, HDL-C, hypertension status (yes/no, defined as according to the 1997 JNC VI criterion [6], education, and smoking exposure (ever smoke [yes/no] and current smoke [yes/no]); (3) repeating the regression analysis in (2) with additional covariate adjustment for the potential mediator of interest; (4) computing the difference in estimated regression coefficients for rs10846744 from the models with and without adjustment for the potential mediator of interest, and (5) using a bias-corrected bootstrap method to assess significance of the mediation effect [7].

Following stratified analyses, we performed meta-analysis to combine results across all four race/ethnic groups. We implemented Z-score based meta-analysis in METAL [8], as our mediation analysis approach provided p-values and effect sizes but not standard errors for input into meta-analysis.

**References**

1. Manichaikul A, Naj AC, Herrington D, Post W, Rich SS, Rodriguez A. Association of SCARB1 variants with subclinical atherosclerosis and incident cardiovascular disease: The multi-ethnic study of atherosclerosis. Arterioscler Thromb Vasc Biol. 2012; doi:10.1161/ATVBAHA.112.249714

2. Manichaikul A, Mychaleckyj J, Rich SS, Daly K, Sale M, Chen W-M. Robust Relationship Inference in Genome Wide Association Studies. Bioinformatics. 2010; doi:10.1093/bioinformatics/btq559

3. Computing RF for S. R Development Core Team. R A Lang Environ Stat Comput. 2011;

4. Chen M, Yang Q. GWAF: an R package for genome-wide association analyses with family data. Bioinformatics. 2010; doi:10.1093/bioinformatics/btp710

5. Genuth S, Alberti K, Bennett P, Buse J, Defronzo R, Kahn R, et al. Follow-up report on the diagnosis of diabetes mellitus. Diabetes Care. 2003; doi:10.2337/diacare.26.12.3331

6. Joint National Committee on prevention evaluation, and treatment of high blood pressure detection. The sixth report of the Joint National Committee on prevention, detection, evaluation, and treatment of high blood pressure. Arch Intern Med. 1997;

7. Fritz MS, MacKinnon DP. Required sample size to detect the mdiated effect. Psychol Sci. 2010; doi:10.1111/j.1467-9280.2007.01882.x.Required

8. Willer CJ, Li Y, Abecasis GR. METAL: Fast and efficient meta-analysis of genomewide association scans. Bioinformatics. 2010; doi:10.1093/bioinformatics/btq340
