# Supplementary material for: Lp-PLA2, scavenger receptor class B type I gene (SCARB1) rs10846744 variant, and cardiovascular disease
Source: PLoS One. 2018 Oct 5;13(10):e0204352. doi: 10.1371/journal.pone.0204352 (PMC6173398; doi:10.1371/journal.pone.0204352)
Supplement: S1 Table — (DOCX) [file pone.0204352.s002.docx]

**S1 Table. Results of associations between *SCARB1* rs10846744 and the primary outcomes within MESA race/ethnic groups.**

| **Main outcome*** | **Group** | **N** | **Beta** | **SE** | ***P*-value** | **log10 Bayes Factor** | **Heterogeneity**  **I-squared /**  **Heterogeneity *P*-value** |
| --- | --- | --- | --- | --- | --- | --- | --- |
| CV-all | Caucasian | 2278 | 0.370 | 0.118 | 0.002 |  |  |
|  | African-American | 1566 | -0.010 | 0.125 | 0.938 |  |  |
|  | Hispanic | 1393 | -0.325 | 0.139 | 0.019 |  |  |
|  | Chinese-American | 690 | 0.317 | 0.214 | 0.139 |  |  |
|  | Meta-analysis |  | 0.077 | 0.069 | 0.263 | 0.63 | 81.6 / 0.001 |
| CVD-confirmed | Caucasian | 2278 | 0.325 | 0.138 | 0.018 |  |  |
|  | African-American | 1566 | 0.089 | 0.144 | 0.535 |  |  |
|  | Hispanic | 1393 | -0.276 | 0.151 | 0.068 |  |  |
|  | Chinese-American | 690 | 0.540 | 0.279 | 0.053 |  |  |
|  | Meta-analysis |  | 0.103 | 0.080 | 0.195 | -0.03 | 73.6 / 0.01 |
| All-cause mortality | Caucasian | 2278 | -0.026 | 0.121 | 0.830 |  |  |
|  | African-American | 1566 | -0.101 | 0.110 | 0.362 |  |  |
|  | Hispanic | 1393 | -0.196 | 0.135 | 0.147 |  |  |
|  | Chinese-American | 690 | -0.250 | 0.201 | 0.214 |  |  |
|  | Meta-analysis |  | -0.117 | 0.066 | 0.076 | 0.06 | 0.0 / 0.71 |
| Common IMT | Caucasian | 2250 | 0.009 | 0.007 | 0.165 |  |  |
|  | African-American | 2406 | 0.007 | 0.005 | 0.230 |  |  |
|  | Hispanic | 1999 | 0.015 | 0.006 | 0.015 |  |  |
|  | Chinese-American | 687 | 0.017 | 0.010 | 0.083 |  |  |
|  | Meta-analysis |  | 0.011 | 0.003 | 0.001 | 1.76 | 0.0 / 0.68 |
| Internal IMT | Caucasian | 2232 | 0.012 | 0.016 | 0.446 |  |  |
|  | African-American | 2385 | 0.003 | 0.013 | 0.793 |  |  |
|  | Hispanic | 1968 | 0.021 | 0.014 | 0.135 |  |  |
|  | Chinese-American | 683 | 0.006 | 0.022 | 0.799 |  |  |
|  | Meta-analysis |  | 0.011 | 0.008 | 0.148 | 0.01 | 0.0 / 0.81 |

Regression models were adjusted for age, sex, study site, PCs of ancestry, BMI, diabetes status, serum creatinine, LDL-C, HDL-C, lipid medication, hypertension status, education, and smoking exposure (ever smoke and current smoke), Genetic association shown for effect allele C (vs. reference allele G). Meta-analysis results were obtained as follows: (a) *P*-values from fixed effects meta-analysis implemented in METAL, (b) log10 Bayes factors in favor of association from trans-ethnic meta-analysis implemented in MANTRA, (c) Heterogeneity I-squared and Heterogeneity *P*-values from Cochran’s Q test as implemented in METAL.
